# Mesostructural Modeling of Dynamic Modulus and Phase Angle Master Curves of Rubber Modified Asphalt Mixture

**DOI:** 10.3390/ma12101667

**Published:** 2019-05-22

**Authors:** Linhao Gu, Luchuan Chen, Weiguang Zhang, Haixia Ma, Tao Ma

**Affiliations:** 1 School of Transportation, Southeast University, Nanjing, Jiangsu 210096, China; gulinhao@seu.edu.cn (L.G.); wgzhang@seu.edu.cn (W.Z.); 2 State Engineering Laboratory of Highway Maintenance Technology, Changsha University of Science and Technology, Changsha 410114, China; 3 Qilu Transportation Development Group, 1 Longaoxi, Jinan, Shandong 200101, China; chenluchuan01@163.com; 4 Shandong Guilu Expressway Construction Co. LTD., 23 Changrun, Liaocheng, Shandong 252000, China; 230129425@seu.edu.cn

**Keywords:** rubber modified asphalt mortar, asphalt mixture, continuous and discrete spectrum, finite element model, dynamic modulus, phase angle, master curves

## Abstract

The main objective of this paper was to develop a mesostructure-based finite element model of rubber modified asphalt mixture to predict both the dynamic modulus master curve and phase angle master curve under a large frequency range. The asphalt mixture is considered as a three-phase material consisting of aggregate, asphalt mortar, and air void. The mesostructure of the asphalt mixture was digitized by a computed tomography (CT) scan and implemented into finite element software. The 2S2P1D model was used to obtain the viscoelastic information of an asphalt mortar under a large range of frequencies and temperatures. The continuous spectrum of the 2S2P1D model was converted to a discrete spectrum and characterized by the generalized Maxwell model for numerical simulation. The Prony series parameters of the generalized Maxwell model and the elastic modulus of the aggregates were inputted into the finite element analysis as material properties. The dynamic modulus tests of a rubber modified asphalt mortar and asphalt mixture were conducted under different temperatures and loading frequencies. The dynamic modulus master curve and phase angle master curve of both asphalt mortar and asphalt mixture were constructed. The frequency of the finite element simulations of the dynamic modulus tests ranged from 10^−6^ to 10^4^. The dynamic modulus and phase angle of the asphalt mixture was calculated and the master curves were compared with the master curves obtained from the experimental data. Furthermore, the effect of the elastic modulus of aggregates on the master curves was analyzed. Acceptable agreement between dynamic modulus master curves obtained from experimental data and simulation results was achieved. However, large errors between phase angle master curves appeared at low frequencies. A method was proposed to improve the prediction of the phase angle master curve by adjusting the equilibrium modulus of the asphalt mortar.

## 1. Introduction

The asphalt mixture exhibits linear viscoelasticity (LVE) under small strain conditions (150 με). The dynamic complex modulus test was introduced by NCHRP 9–19 to characterize the viscoelastic mechanical behavior of asphalt mixture. The dynamic modulus and phase angle master curves can be constructed by employing the time-temperature superposition principle (TTSP) [1,2,3]. However, laboratory tests are time-consuming and require expensive advanced testing equipment. In the past few decades, scholars have been working on virtual test methods to obtain mechanical properties more efficiently [4,5,6,7,8]. Meanwhile, with the development of CT technology and digital image processing, virtual test simulations based on the mesostructure or microstructure model were conducted using a numerical algorithm like the finite element model and discrete element model [9,10,11,12,13,14]. You [15] employed DIC (digital image correlation) to reconstruct a mechanical model of the asphalt mixture. A two-dimensional (2D) clustered DEM (discrete element method) model was built to simulate the dynamic modulus test of sand mastic and asphalt mixture. You and Dai [16,17] used a micromechanical-based FEM (finite element method) model to simulate the dynamic modulus of the hot mix asphalt mixture. A two-phase model consisting of aggregates and sand mastic was modeled with finite element method. The results had good agreement with that of the DEM models. Dai [18] employed the same procedure to predict both the dynamic modulus and phase angle of the stone-based composites of the asphalt mixture. Ying et al. [19] reconstructed a 3D heterogeneous FEM model by X-ray tomography images. Dynamic modulus test simulations were conducted. It indicated that the mastic had more influence on the dynamic modulus than the aggregates. Liu and You [20] analyzed the creep stiffness of the three-dimensional heterogeneous structure of the asphalt mixture. It was found that the geometric characteristics of aggregate orientation and sphericity have a certain comprehensive influence on the creep stiffness of the mixture. Chen proposed a random packing algorithm to generate a 3D virtual asphalt mixture sample called RAGS (random aggregate structures). The dynamic modulus and phase angle were simulated using both the 2D and 3D finite element model. It was shown that the 2D model underestimates the dynamic modulus while overestimating the phase angle. Cao [6] generated 2D FEM models of asphalt mixture by random packing. A large number of simulations were conducted and a theoretical statistic formula for describing the size effort of a dynamic modulus was proposed. 

The studies mentioned above mainly focused on the prediction of a dynamic modulus at one temperature, which means in a small frequency range. In order to obtain the full viscoelastic information of an asphalt mixture, the prediction of a dynamic modulus and phase angle master curves under a wide range of frequency needs to be studied.

Many attempts were made to obtain master curves of an asphalt mixture from mechanical properties of an asphalt mortar or asphalt binder. Olard [21] proposed a relationship between the binder and the mixture complex modulus. The dynamic modulus of the asphalt mixture can be calculated using the glassy modulus and equilibrium modulus of both the asphalt mixture and asphalt binder. Kuna [22] proposed a method to construct stress dependent master curves of foamed bitumen treated mixes (FBMs). The laboratory data were shifted both horizontally and vertically according to the TTSP to create a single master curve. Blasl [23] employed the Christensen and Anderson model (CAM) and the Olard-Di Benedetto model (2S2P1D) to characterize the master curves of the bitumen samples extracted from stone mastic asphalt mixtures. Different objective functions were used to fit the master curves, but the master curves of the asphalt mixture were not predicted. Nobakht [24] proposed a kinetic-based aging model to predict the dynamic modulus master curve of asphalt mixtures. The influence of aging on the dynamic modulus and phase angle master curves was analyzed. Yin [25] conducted dynamic modulus tests of four asphalt binders and corresponding asphalt mixtures. The dynamic modulus master curves were constructed using both the Williams–Landel–Ferry (WLF) equation and the Arrhenius equation. It can be seen that the relationship between the master curves of the asphalt mixture and asphalt mortar was not fully studied. In these studies, the influence of the mesostructure of an asphalt mixture on the dynamic modulus and phase angle was not considered.

In this study, dynamic modulus test simulations using a mesostructure-based finite element model were conducted under a large frequency range to calculate the dynamic modulus and phase angle of asphalt mixture. To be able to accurately predict the master curves of the asphalt mixture, the mechanical behavior of the asphalt mixture and asphalt mortar under a wide range of frequency needs to be accurately characterized by viscoelastic constitutive models.

In the past decades, many viscoelastic constitutive models have been proposed. The generalized Maxwell model (GMM) was widely used to characterize the viscoelastic mechanical behavior of asphalt concretes. It consists of a combination of springs and linear dashpots and is known as the discrete spectrum model. The relaxation models in the time domain can be expressed by the Prony series, which can be analytically converted into the frequency domain and conveniently implemented into the hereditary integrals of numerical simulation [1,3,26]. However, the determination of model parameters is a big problem for the generalized model, especially when employed to characterize the dynamic modulus and phase angle master curves of the asphalt mixture. The number of relaxation times in the discrete spectrum needs to be large enough to cover a wide range in the frequency domain to ensure accuracy. Since the fitting process needs to solve a set of ill-posed nonlinear equations, a large number of parameters will cause severe difficulties in the numerical calculation [27,28,29,30,31].

Other than discrete spectrum models, the continuous spectrum models can characterize the dynamic modulus and phase angle master curves in a wide frequency range with few parameters. The continuous spectrum model is usually expressed by a complex function and can represent the dynamic modulus and phase angle simultaneously. A lot of continuous spectrum models have been proposed. Superpave-A-357 proposed a creep equation in the form of a power law function to characterize the viscoelastic mechanical behavior of asphalt concretes. Zeng [32], based on the CAM (Christensen–Anderson–Marasteanu) constitutive model of an asphalt binder [33], proposes a model for characterizing the master curve and phase angle master curve of the dynamic shear modulus of asphalt concrete. Olard [21] proposed a continuous spectrum model for asphalt concrete based on the Huet model and Huet–Sayegh (HS) model. The 2S2P1D model can accurately describe the mechanical behavior of the asphalt concrete over the entire frequency range and temperature range. Havriliak and Negami [34] proposed the HN model based on the Cole–Cole model and Davison–Cole model. However, the numerical implementation is difficult for the continuous spectrum model since the analytical expression of the relaxation modulus in the time domain does not exist.

The main objective of this study is to propose a method to predict the dynamic modulus and phase angle master curves of rubber modified asphalt mixture. The continuous spectrum model is used to capture the viscoelastic information of an asphalt mortar and then converted into a discrete spectrum model for finite element implementation. Simulations with a large frequency range are conducted and the dynamic modulus and phase angle master curves are calculated. The effect of the aggregate modulus on the dynamic modulus and phase angle of the asphalt mixture is studied. The prediction of the phase angle is improved by adjusting the equilibrium modulus of the asphalt mortar.

## 2. Theoretical Background

### 2.1. Continuous Spectrum Model for an Asphalt Mortar and Asphalt Mixture 

The rheological models consisting of a combination of linear springs and linear dashpot can characterize the viscoelastic behavior of an asphalt binder, asphalt mortar, and asphalt mixture. The relaxation modulus and creep compliance are commonly represented by the generalized Maxwell model and generalized Kevin–Voigt model, which can be expressed by the Prony series and Dirichlet series, respectively.

The constitutive relation for linear viscoelastic materials can be written as a convolution integral:

(1) $$\sigma \left(t\right)=\int_{0}^{t}E\left(t-\xi \right)\frac{d\varepsilon }{d\xi }d\xi$$

where σ is stress; E is the relaxation modulus; ε is the strain; *t* is time; and ξ is the integral variable.

The relaxation modulus of the generalized Maxwell model is written as:

(2) $$E\left(t\right)=E_{e}+\sum_{i=1}^{n}E_{i}e^{-t/\tau_{i}}$$

where $E_{e}$ is called the equilibrium modulus, which is the relaxation modulus when the time approaches infinity; $E_{i}$ and $\tau_{i}$ are the relaxation modulus and the relaxation time of the *i*th Maxwell model; and *n* is the number of the sub-models. 

The set of $E_{i}$ and $\tau_{i}$ is called the discrete spectrum, which represents the distribution of relaxation modulus over relaxation time. When the number of the sub-models increases to infinity and the interval between relaxation time decreases to zero, the Prony series becomes an integral and the discrete spectrum becomes the continuous spectrum. The relaxation modulus function expressed by a continuous spectrum model is:

(3) $$E\left(t\right)=E_{e}+\int_{0}^{\infty }\frac{H\left(\xi \right)}{\xi }e^{-t/\xi }d\xi$$

where H(ξ) is the continuous spectrum function. 

The relaxation modulus represents the viscoelastic behavior in the time domain. It can be converted into the complex modulus to represent the viscoelastic behavior in the frequency domain by the Laplace transform. The complex modulus, storage modulus, and loss modulus can be expressed as:

(4) $$E^{*}\left(i\omega \right)=L\left\{E\left(s\right)\right\}|{}_{s=i\omega }=E^{\prime }\left(i\omega \right)+iE^{\prime \prime }\left(i\omega \right)$$

(5) $$E^{\prime }\left(i\omega \right)=E_{e}+\int_{0}^{\infty }\frac{H\left(\xi \right)}{\xi }\frac{\omega^{2}\xi^{2}}{1+\omega^{2}\xi^{2}}d\xi$$

(6) $$E^{\prime \prime }\left(i\omega \right)=\int_{0}^{\infty }\frac{H\left(\xi \right)}{\xi }\frac{\omega \xi }{1+\omega^{2}\xi^{2}}d\xi$$

where $E^{*}$ is the complex modulus; L is the Laplace transform; s is the Laplace transform variable; $E^{\prime }$ is the storage modulus; $E^{\prime \prime }$ is the loss modulus; and ω= 2πf is the angular frequency, and *f* is the frequency.

**Figure 1:**
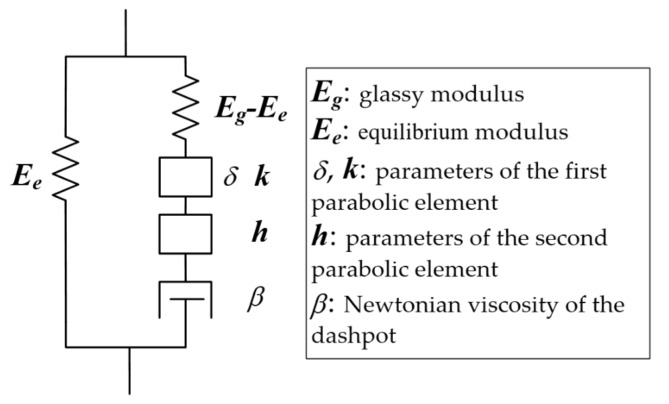
Phenomenological structure of the 2S2P1D model.

The 2S2P1D model was proposed to characterize the viscoelastic properties of both the asphalt binder and asphalt mixture. It is expressed by a complex function and can characterize the dynamic modulus and phase angle simultaneously. It consists of two springs, two parabolic elements, and one dashpot. The phenomenological structure of the 2S2P1D model is shown in Figure 1.

The parabolic element, also called the Abel model, can be seen as a rheological element between the linear spring and the linear dashpot. Its constitutive relation in the time domain can be expressed by a fractional order derivation, while the constitutive relations of the spring and the dashpot are zero order and first order respectively. 

The parabolic element is defined as:

(7) $$\mathrm{Creep}\;\mathrm{compliance}:\;J\left(t\right)=\delta {\left(\frac{t}{\tau_{ref}}\right)}^{k}$$

(8) $$\mathrm{Complex}\;\mathrm{modulus}:\;E^{*}\left(i\omega \right)=\frac{{\left(i\omega \tau_{ref}\right)}^{k}}{\delta \;\Gamma \left(k+1\right)}$$

where δ and k are material constants; ω is the angular frequency; Γ is the Gamma function; and $\tau_{ref}$ is the characteristic time that accounts for the time-temperature superposition principle and determined by the WLF (Williams–Landel–Ferry) equation:

(9) $$\tau \left(T\right)=\alpha_{T}*\tau_{ref}$$

where $\alpha_{T}$ is the shift factor at temperature *T*; and $\tau_{ref}$ is the characteristic time at the reference temperature.

$\alpha_{T}$ is determined by the WLF equation:

(10) $$\log\left(\alpha_{T}\right)=\frac{-C_{1}\left(T-T_{ref}\right)}{C_{2}+\left(T-T_{ref}\right)}$$

where $C_{1}$ and $C_{2}$ are constants of the WLF equation; and $T_{ref}$ is the reference temperature.

According to the phenomenological structure, the complex modulus function of the 2S2P1D model can be easily derived in the Laplace domain:

(11) $$E^{*}\left(i\omega \right)=E_{e}+\frac{E_{g}-E_{e}}{1+\delta {\left(i\omega \tau_{ref}\right)}^{-k}+{\left(i\omega \tau_{ref}\right)}^{-h}+{\left(i\omega \tau_{ref}\beta \right)}^{-1}}$$

where $E_{e}$ is the equilibrium modulus; $E_{g}$ is the glassy modulus or instantaneous modulus, which is the relaxation modulus when time equals zero; ω is the angular frequency; δ, k, and h are parameters of the parabolic elements and set as 0 < k < h < 1; β is the Newtonian viscosity of the dashpot; and $\tau_{ref}$ is the characteristic time.

### 2.2. Determination of the Discrete Spectrum

As mentioned above, the continuous spectrum models can characterize the viscoelastic behavior of the asphalt concrete in the frequency domain more accurately and effectively. The continuous spectrum models can capture the viscoelastic information under a large range of temperatures and frequencies with fewer model parameters than that of the generalized Maxwell model. The accuracy of the generalized Maxwell model can be improved by increasing the number of sub-models. However, when parameters of the generalized Maxwell model are directly obtained from the dynamic modulus test, the fitting process needs to solve a set of ill-posed nonlinear equations. As the number of parameters increases, the difficulty of fitting calculation increases.

The continuous spectrum models have disadvantages in numerical calculation and finite element analysis. Some continuous spectrum models, like the HS model and the 2S2P1D model, do not have an analytical expression of the relaxation modulus in the time domain. Other continuous spectrum models, like the generalized fractional Maxwell model, can be analytically converted into a relaxation modulus using the Mittag–Leffler function [35]. In this case, the incremental strain history and incremental time history need to be stored during the numerical calculation, which requires a large storage space and decreases computational efficiency. The generalized Maxwell model expressed by the Prony series, however, can be conveniently written as an incremental form in the finite element algorithm. Only the incremental strain and incremental time of the last incrementation need to be stored, so the generalized Maxwell model is more suitable for finite element analysis.

In this paper, the 2S2P1D model is used to capture the viscoelastic information of the asphalt mixture from dynamic modulus test data and then the continuous spectrum is converted into a discrete spectrum in the form of Prony series for finite element implementation.

The continuous spectrum function of a continuous spectrum model can be derived by the following formula [3]:

(12) H(τ)=±1πImE∗(1τe±iπ)

where Im denotes the imaginary part of the complex number; $E^{*}$ is the complex modulus function; and τ is relaxation time.

The continuous spectrum function of the 2S2P1D model is:

(13) $$H\left(\tau \right)=\frac{E_{g}}{\pi \sqrt{A^{2}+B^{2}}}\sin\varphi$$

where

$A=1+\delta {\left(\frac{\tau_{ref}}{\tau }\right)}^{-k}\cos k\pi +{\left(\frac{\tau_{ref}}{\tau }\right)}^{-h}\cos h\pi -{\left(\frac{\tau_{ref}}{\tau }\beta \right)}^{-1}$;
$B=\delta {\left(\frac{\tau_{ref}}{\tau }\right)}^{-k}\sin k\pi +{\left(\frac{\tau_{ref}}{\tau }\right)}^{-h}\sin h\pi$;
$\varphi =\arctan\frac{B}{A}$.

By interpreting the integral in Equation (3) as discrete approximations, the relaxation modulus function can be written as:

(14) $$\begin{array}{ll} E\left(t\right) & =E_{e}+\int_{0}^{\infty }\frac{H\left(\xi \right)}{\xi }e^{-t/\xi }d\xi \\ & =E_{e}+\sum_{i=1}^{n}\left[\frac{H\left(\tau_{i}\right)}{\tau_{i}}\cdot \Delta \tau_{i}\right]e^{-t/\tau_{i}} \end{array}$$

where the set of $\tau_{i}$ is discrete relaxation times as mentioned in Equation (2); and $\Delta \tau_{i}$ is the interval between relaxation times.

Comparing Equation (14) with Equation (2), the discrete spectrum can be determined as follow:

(15) $$E_{i}=\frac{H\left(\tau_{i}\right)}{\tau_{i}}\cdot \Delta \tau_{i}$$

where $E_{i}$ is the discrete relaxation modulus as mentioned in Equation (2).

The set of relaxation time $\tau_{i}$ needs to be artificially selected. When the relaxation times are evenly distributed on the time axis, the convergence of the discrete spectrum to the continuous spectrum is very slow [36]. The convergence can be greatly improved by evenly distributing the relaxation times on the logarithmic time axis. In this case, Equation (3) and Equation (15) need to be rewritten as:

(16) $$E\left(t\right)=E_{e}+\int_{-\infty }^{\infty }H\left(\xi \right)e^{-t/\xi }d\ln\xi$$

(17) $$E_{i}=H\left(\tau_{i}\right)\cdot \Delta \ln\tau_{i}$$

The details of the parameter calibration of the 2S2P1D model and the determination of the discrete spectrum will be elaborated in the next section.

## 3. Experiment and Parameter Acquisition

### 3.1. Dynamic Modulus Tests of the Asphalt Mortar and Asphalt Mixture

In this study, dynamic modulus tests of both the asphalt mortar and asphalt mixture were conducted. The test data of the asphalt mortar were used to obtain the parameters of the 2S2P1D model and the generalized Maxwell model. The test data of the asphalt mixture were used to verify the finite element simulation.

**Figure 2:**
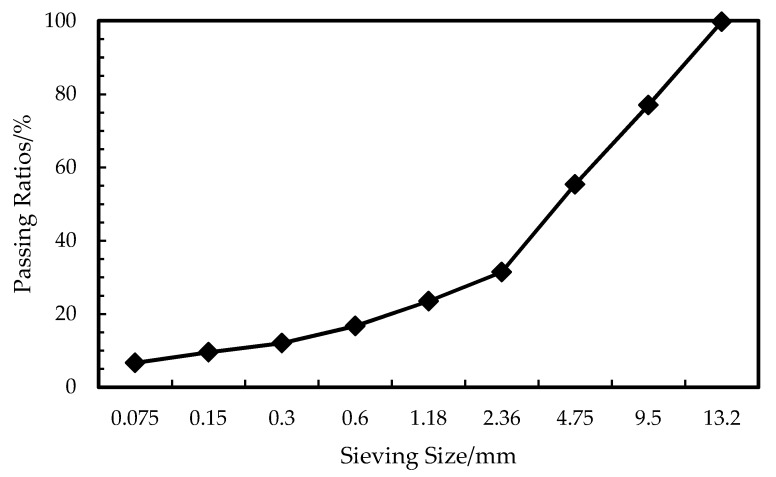
Aggregate gradation of the asphalt mixture.

The asphalt mixture was dense graded with a 13.2 mm nominal maximum aggregate size. The gradation is shown in Figure 2.

The asphalt binder used was a rubber modified asphalt. The asphalt content was 4.5% by weight and the air void is 3.1%.

The gradation of the asphalt mortar was the same as that of asphalt mixture finer than 2.36 mm. The asphalt content of the asphalt mortar was calculated based on the specific surface area of the aggregates. It was assumed that the asphalt was uniformly dispersed and attached to the surface of the aggregates. The amount of asphalt coated on the aggregates was proportional to the specific surface area of the aggregate. Table 1 shows the specific surface area of aggregates with different size. Table 2 shows the calculation of the asphalt content of asphalt mortar.

The superpave gyratory compactor (SGC) was utilized to compact specimens with 150 mm diameter and 170 mm height. The asphalt mixture samples were cut into a cylinder with 100 mm diameter and 100 mm height. The asphalt mortar samples were cut into a smaller cylinder with 50 mm diameter and 50 mm height to reduce the effect of potential plastic deformation at high temperatures.

The dynamic modulus tests of the asphalt mixture were conducted under four temperatures and six loading frequencies. The test temperatures were −10 °C, 10 °C, 30 °C, and 50 °C and the loading frequencies were 0.1 Hz, 0.5 Hz, 1 Hz, 5 Hz, 10 Hz, and 20 Hz.

The dynamic modulus tests of the asphalt mortar were conducted under five temperatures and nine loading frequencies. The test temperatures were −10 °C, 0 °C, 10 °C, 20 °C, and 30 °C and the loading frequencies were 0.1 Hz, 0.2 Hz, 0.5 Hz, 1 Hz, 2 Hz, 5 Hz, 10 Hz, 20 Hz, and 25 Hz.

All the dynamic modulus tests were performed under stress-control conditions using a UTM-25 testing machine (IPC Global, Melbourne, VIC, Australia). Teflon film was used to reduce the friction between the sample and the indenter.

The dynamic modulus is the absolute value of the complex modulus:

(18) $$\left|E^{*}\right|=\sqrt{{\left(E^{\prime }\right)}^{2}+{\left(E^{\prime \prime }\right)}^{2}}$$

It can be determined by calculating the ratio of stress amplitude and strain amplitude at a steady state of the dynamic modulus test, which is the average value of the last five cycles according to the Chinese specification JTG E20-2011 [37].

(19) $$\left|E^{*}\right|=\frac{\sigma_{0}}{\varepsilon_{0}}$$

where $\sigma_{0}$ is the stress amplitude; and $\varepsilon_{0}$ is the strain amplitude.

Phase angle is the argument of the complex modulus and determined by the average time delay of the last five cycles:

(20) $$\phi =\left(\frac{T_{i}}{T_{p}}\right)\times 360$$

where $T_{i}$ is the time delay; and $T_{p}$ is the sinusoidal load cycle.

The number of parallel samples is required larger than three by the Chinese specifications and the number of samples tested in this study is four. The dynamic modulus and phase angle are calculated using the following equation based on the T-distribution.

(21) $$\left|E^{*}\right|=\bar{\left|E^{*}\right|}-t\times \frac{S_{E}}{\sqrt{n}}$$

(22) $$\phi =\bar{\phi }-t\times \frac{S_{\phi }}{\sqrt{n}}$$

where $\bar{\left|E^{*}\right|}$ and $\bar{\phi }$ are the average dynamic modulus and phase angle of parallel samples; $S_{E}$ and $S_{\phi }$ are the standard deviation of dynamic modulus and phase angle; *n* is the number of parallel samples; and *t* is the parameter of the T-distribution and determined according to the number of samples and the confidence rate. When the number of samples is four and the confidence rate is 95%, *t* equals 2.354.

### 3.2. Parameter Acquisition

Seven parameters of the 2S2P1D model and two parameters of the WLF equation need to be determined. Equations (9), (10), and (11) were utilized to fit the dynamic modulus test data in the frequency domain. A nonlinear minimization algorithm using differential evolution method was performed on the target error function *F* as follow:

(23) $$\min F\left(E_{e},E_{g},\delta ,k,h,\beta ,\tau_{ref},C_{1},C_{2}\right)=\frac{1}{N}\left(\sqrt{{\sum_{i=1}^{N}\left(1-\frac{\left|E_{c,i}^{*}\right|}{\left|E_{t,i}^{*}\right|}\right)}^{2}}+\sqrt{{\sum_{i=1}^{N}\left(1-\frac{\phi_{c,i}}{\phi_{t,i}}\right)}^{2}}\right)$$

where $\left|E_{c,i}^{*}\right|$ and $\left|E_{t,i}^{*}\right|$ are the calculated dynamic modulus and test dynamic modulus, respectively; $\phi_{c,i}$ and $\phi_{t,i}$ are the calculated phase angle and test phase angle, respectively; and *N* is the number of the test data points at all frequencies.

The fitting procedure is conducted using the Mathematica software package. Table 3 lists the calculated model parameters of the asphalt mortar at a reference temperature of 0 °C.

**Figure 3:**
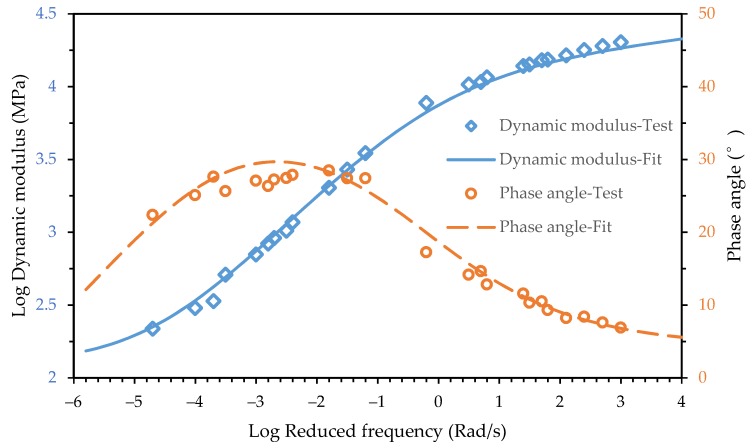
Test data and fitting curve of the asphalt mixture for the dynamic modulus and phase angle.

**Figure 4:**
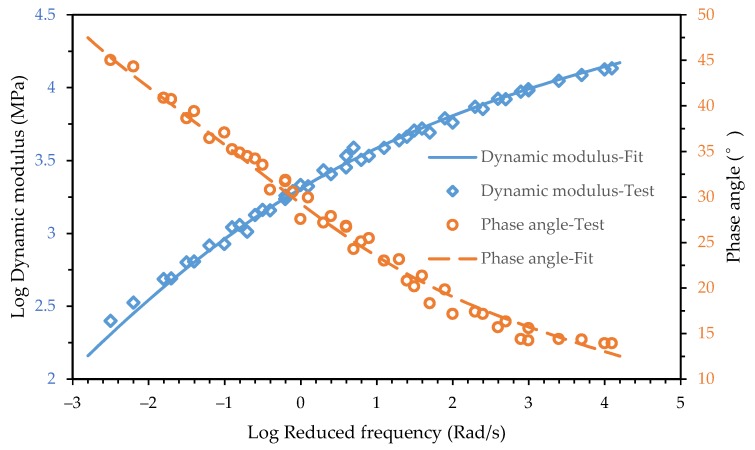
Test data and fitting curve of the asphalt mortar for the dynamic modulus and phase angle.

The fitting result is shown in Figure 3 and Figure 4. It shows that the 2S2P1D model can characterize the dynamic modulus and the phase angle master curves of both the asphalt mixture and asphalt mortar accurately.

Comparing the phase angle main curve of the asphalt mixture and asphalt mortar in Figure 3 and Figure 4, it can be seen that the phase angle of the asphalt mixture and asphalt mortar was significantly different at low frequencies. The phase angle of the asphalt mixture decreased when the reduced frequency approached zero and infinity and peaked at 10^−3^ Rad/s while the phase angle of the asphalt mortar decreased as the reduced frequency decreased. The maximum phase angle of the asphalt mixture is 30°, which indicates that the storage modulus accounts for a larger proportion of dynamic modulus. Due to the inconvenience of conducting the dynamic modulus test for an asphalt mortar at high temperatures, the minimum reduced frequency of test data in Figure 4 only reached 10^−3^ Rad/s but the corresponding phase angle already reached 45°. With the decrease of the reduced frequency, the phase angle of the asphalt mortar would keep increasing. The loss modulus would become larger than the storage modulus.

As mentioned above, to convert the continuous spectrum into the discrete spectrum efficiently, the relation times are uniformly distributed on the logarithmic time axis, so Equation (17) can be rewritten as:

(24) $$E_{i}=H\left(\tau_{i}\right)\cdot \Delta \ln10^{1/n}$$

where *n* is the number of relaxation time $\tau_{i}$ per decade.

**Figure 5:**
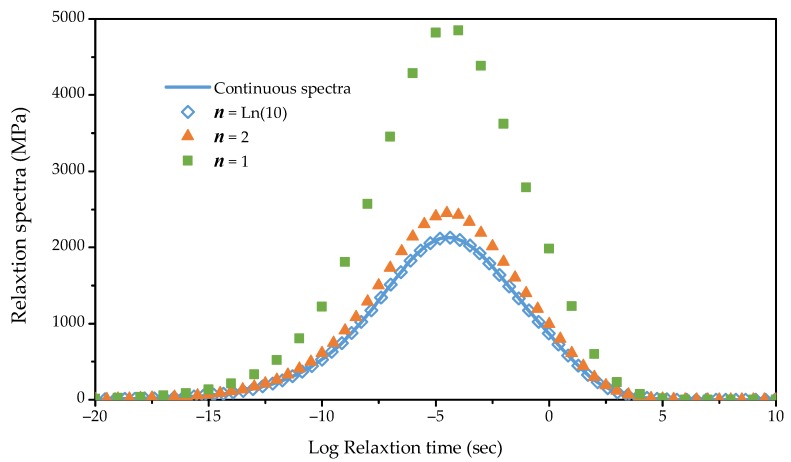
Continuous spectrum and discrete spectrum with different values of *n*.

According to Equations (13) and (17), the continuous spectrum function and the discrete spectrum can be calculated. Figure 5 shows the continuous spectrum and discrete spectrum with different values of *n*. It can be seen that as *n* increased, the difference between the discrete spectrum and the continuous spectrum curve decreased. When n =ln(10) and $\Delta \ln\tau_{i}\;=\;1$, the discrete spectrum curve coincides with the continuous spectrum curve, so n=ln(10) was used to calculate the discrete spectrum.

Since $\Delta \tau_{i}$ was obtained, the set of $\tau_{i}$ could be calculated once the range of $\tau_{i}$ on the relaxation time axis is chosen. As shown in Figure 5, the range was from 10^−20^ to 10^10^. It covered the reduced frequency range of the dynamic modulus tests shown in Figure 4. Additionally, the continuous spectrum H(τ) was less than 1 MPa outside the range and was considered small enough to be ignored. The details of the Prony series is listed in Appendix A.

**Figure 6:**
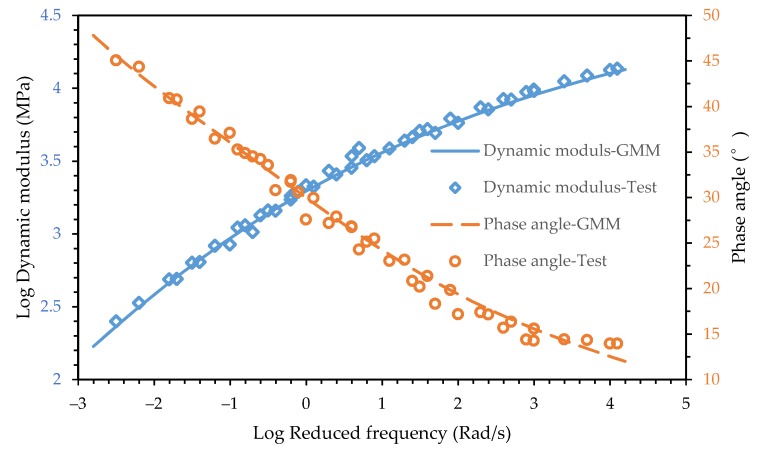
Dynamic modulus and phase angle master curve of the calculated generalized Maxwell model.

To verify the effectiveness of the conversion from the continuous spectrum to the discrete spectrum, the dynamic modulus and phase angle master curves of the calculated generalized Maxwell model were compared with the experimental data, as shown in Figure 6. It can be seen that the master curves had good agreement with the experimental data, which means the conversion was effective. The relative error between the master curves and the test data in Figure 6 was 2.53%, which was also calculated using Equation (23). It was slightly larger than the relative error of 2S2P1D but acceptable.

## 4. Construction of the Finite Element Model

### 4.1. Mesostructure of the Asphalt Mixture 

**Figure 7:**
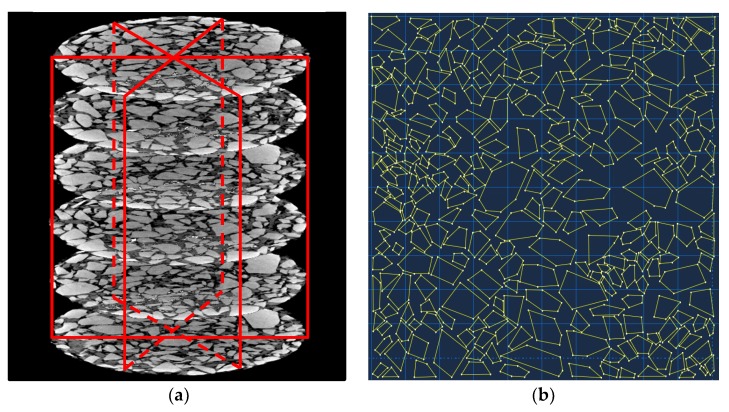
Mesostructure of the asphalt mixture: (**a**) Vertical section of the mixture sample; (**b**) aggregate skeleton sketch in ABAQUS.

The mixture sample with test data closest to the average value of four parallel samples was chosen to be scanned by an X-Ray CT. A set of two-dimensional scan images of the horizontal sections perpendicular to the cylinder axis was first captured. These two-dimensional images were manipulated by image processing software and reconstructed into a three-dimensional mesostructured as shown in Figure 7a. 

Many studies were devoted to analyzing the differences between 3D models and 2D models in simulating the dynamic modulus. In general, the 3D models have higher accuracy while the 2D models tend to underestimate the dynamic modulus and overestimate phase angles [5]. The differences between 3D models and 2D models are related to both the contrast between the inclusions and matrix complex modulus and the contrast between the surface filling rate in 2D models and the volumetric filling rate in 3D models. Firstly, as the modulus contrast between the inclusions and matrix reduces, the difference between models reduces. The modulus contrast between the inclusions and matrix of mastic, mortar, and mixture is studied [38]. It indicates the asphalt mixture has the smallest modulus contrast. Secondly, the filling rate of 2D models and 3D models of the asphalt mixture is very close. Additionally, the element number of 2D models in the following simulation is larger than 20,000. The use of 3D models will dramatically increase the element number and reduce the efficiency of numerical simulation. Based on the above reasons, only 2D models were used in this study.

Three vertical sections of the mixture sample were obtained from the 3D mesostructure, as shown in Figure 7a. The image processing techniques were employed to identify the aggregate skeleton of the 2D image. The coordinate information of aggregate polygon was saved as input files(*.sat), which can be implemented into the finite element software. The aggregate skeleton sketch in ABAQUS is shown in Figure 7b.

### 4.2. Finite Element Model

**Figure 8:**
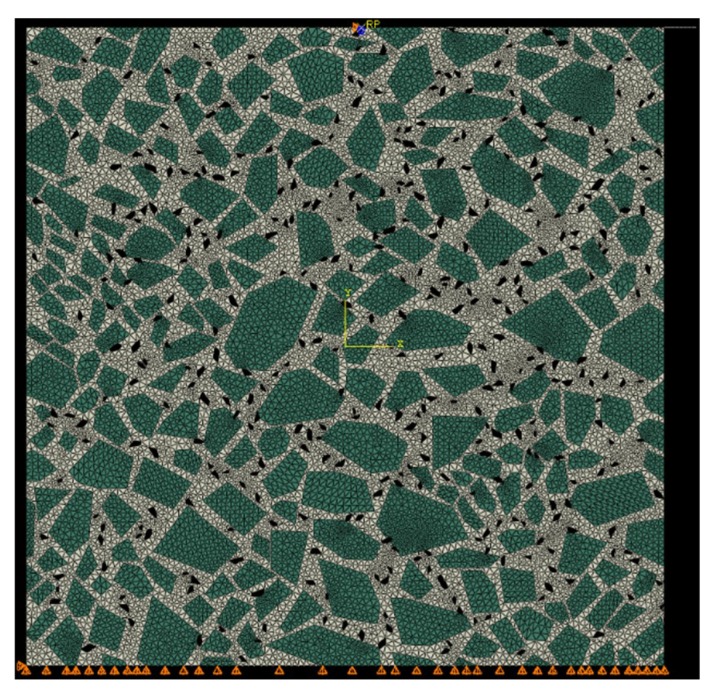
Finite element model of the asphalt mixture.

The size of the 2D finite element model was the same as that of the asphalt mixture test sample, with 100 mm width and 100 mm height, as shown in Figure 8. The element type was CPS8R, an eight-node biquadratic plane stress quadrilateral, reduced integration. The element number was 23,185.

The definition of the constraints was on the basis of the real test conditions. The vertical displacement of the bottom edge was restraint. In order to avoid a large lateral displacement of the model, the nodes in the lower left corner were fixed. A discrete rigid wire was used to represent the indenter of the testing machine to simulate a more realistic stress-control loading condition. The horizontal displacement and the rotation of the indenter was restraint and only the vertical displacement was allowed. The normal behavior of the contact between the sample and the indenter was pressure-overclosure relationships. The tangential behavior was considered frictionless since Teflon film was used in the laboratory test to largely reduce friction between the sample and the indenter. Considering the lateral deformation of the sample under the compressive load, the rigid wire was longer than the top edge of the sample to avoid numerical problems in the contact analysis.

A haversine concentrate force was applied to the reference point of the rigid wire to simulate the stress-control loading condition. The magnitude of the concentrated force was calculated by multiplying the loading stress of the tests by the area of the sample.

The material properties of the asphalt mortar are given in Appendix A. The elastic modulus of aggregates, 58,413 MPa, was obtained from the uniaxial compression test.

Visco Step with the implicit algorithm was adopted. The number of load cycles at each frequency is required in Chinese specification JTG E20-2011. The displacement of the rigid wire was read from the simulation results and then converted into the strain of the mixture sample. The average strain amplitude of the last five cycles was used to calculate the dynamic modulus.

The increment size affects the accuracy of the simulation results especially the phase angle. The error of the phase angle can be written as:

(25) $$\mathrm{Error}\;\mathrm{of}\;\mathrm{the}\;\mathrm{phase}\;\mathrm{angle}\;=\pm \frac{360^{\circ }}{2N_{\mathrm{inc}}}$$

where $N_{\mathrm{inc}}$ is the number of incrementations in one cycle. 

Automatic incrementation size was set with a max size of 1/500 cycle, which ensured that $N_{\mathrm{inc}}$ was greater than 500 and the error of the phase angle was less than 0.36°.

All three 2D mesostructures of the asphalt mixture sample were used in the following simulations. The average value of the predicted dynamic modulus and phase angle from different mesostructures were presented as the simulation results. 

## 5. Simulation Results and Analysis

### 5.1. Simulations of Dynamic Modulus and Phase Angle Master Curves 

**Figure 9:**
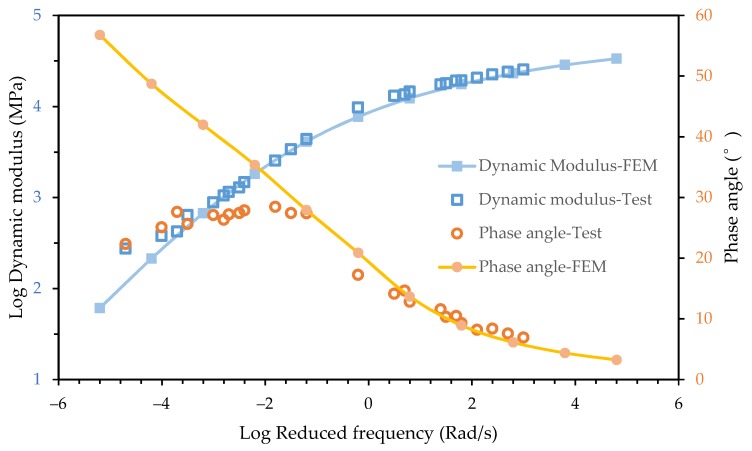
Comparison of simulation results and test data.

The loading frequencies of the dynamic modulus test simulations ranged from 10^−6^ to 10^4^ with an interval of 0.5 on the logarithmic frequency axis. The predicted dynamic modulus and phase angle is plotted in Figure 9, together with the experimental data. It is shown that the predicted dynamic modulus master curve was consistent with experimental data over the entire frequency range, while the magnitude of dynamic modulus was slightly less than the test results. As discussed in the previous section, the 2D finite element models tend to underestimate the dynamic modulus due to neglect the geometric characteristics of aggregates in the third dimension.

However, the predicted phase angle master curve was not consistent with experimental data over the entire frequency range. Good agreement was achieved between the predicted phase angle and the test data when frequency was larger than 0.1 Rad/s. A significant difference appeared as the frequency decreased. In previous studies, the frequency range considered in simulations was usually from 0.1 Hz to 25 Hz as the frequency range of the laboratory tests. It can be seen in Figure 9 that the simulation results and test data of the phase angle in this frequency range were in a good agreement. The significant difference at lower frequency range was ignored.

In the frequency domain, the phase angle master curve of a viscoelastic liquid and a viscoelastic solid was different. When the frequency approached infinity, both the viscoelastic liquid and viscoelastic solid exhibited pure elastic mechanical behavior, which means the phase angle approached zero. When the frequency approached zero, the viscoelastic liquid showed pure viscous mechanical behavior while the viscoelastic solid still showed pure elastic mechanical behavior [3]. In general, the phase angle of a viscoelastic liquid monotonically decreased as the frequency increased while the phase angle master curve of a viscoelastic solid exhibited a bell shape. It can be seen in Figure 4 that the phase angle of the asphalt mortar showed the mechanical behavior of a viscoelastic liquid.

In the finite element simulation, the viscoelastic mechanical behavior was represented only by asphalt mortar since the aggregates were considered pure elastic. Therefore, as shown in Figure 9, the viscoelastic mechanical response of the mesostructure was consistent with the asphalt mortar and the difference of rheology properties between the asphalt mixture and asphalt mortar caused a significant error in predicting phase angle master curves.

### 5.2. Influence of Elastic Modulus of Aggregates on Master Curves 

The elastic modulus of the aggregates used in the previous simulations was obtained in the uniaxial compressive test, but in many studies, the elastic modulus is assumed to range from 50 GPa to 70 GPa and is artificially preselected. The influence of the elastic modulus of aggregate on the dynamic modulus and phase angle master curve needs to be analyzed. 

**Figure 10:**
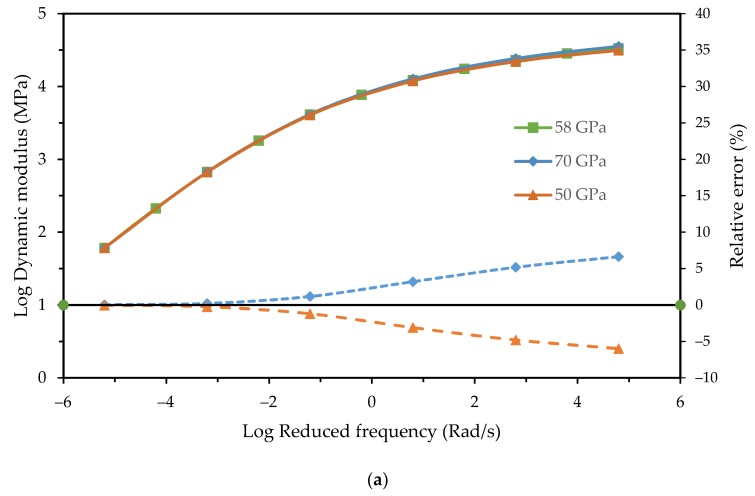

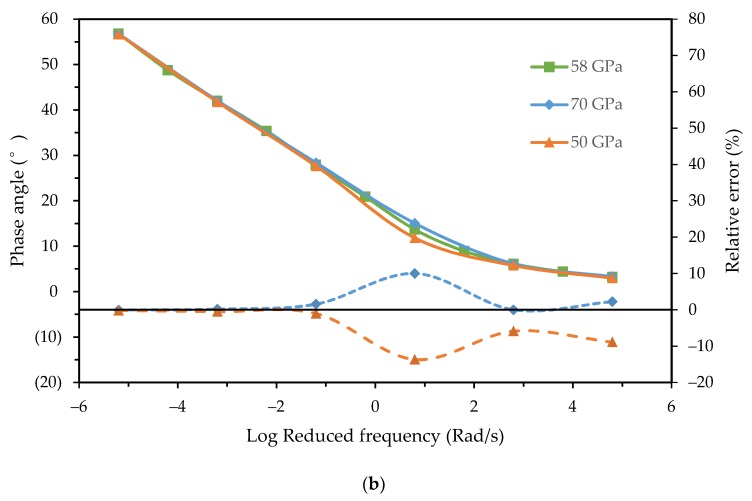
Influence of the elastic modulus of aggregate: (**a**) Dynamic modulus; and (**b**) phase angle.

Three different elastic moduli of aggregates were considered. The loading frequencies were the same as simulations in Section 5.1. The results are plotted in Figure 10.

It shows that the elastic modulus of aggregate had an effect on both the dynamic modulus and phase angle. As the frequency increased, the effect of the modulus on the dynamic modulus monotonically increased while the relative error of the phase angles had fluctuated and reached a peak at 1 Rad/s. The relative error of the dynamic modulus test was less than that of the phase angle. It indicates that the elastic modulus of aggregate had a greater effect on the phase angle.

### 5.3. Improved Prediction of the Phase Angle Master Curve

As discussed in Section 5.1, the finite element simulation cannot accurately predict the phase angle at low frequencies. To solve this problem, a new attempt was made in this study by modifying the equilibrium modulus of the asphalt mortar. 

The equilibrium modulus $E_{e}$ of the asphalt mortar in the 2S2P1D model was very close to zero. The equilibrium modulus of the asphalt mixture, however, could be calibrated using a similar procedure given in Section 3.2 and was 127 Mpa.

**Figure 11:**
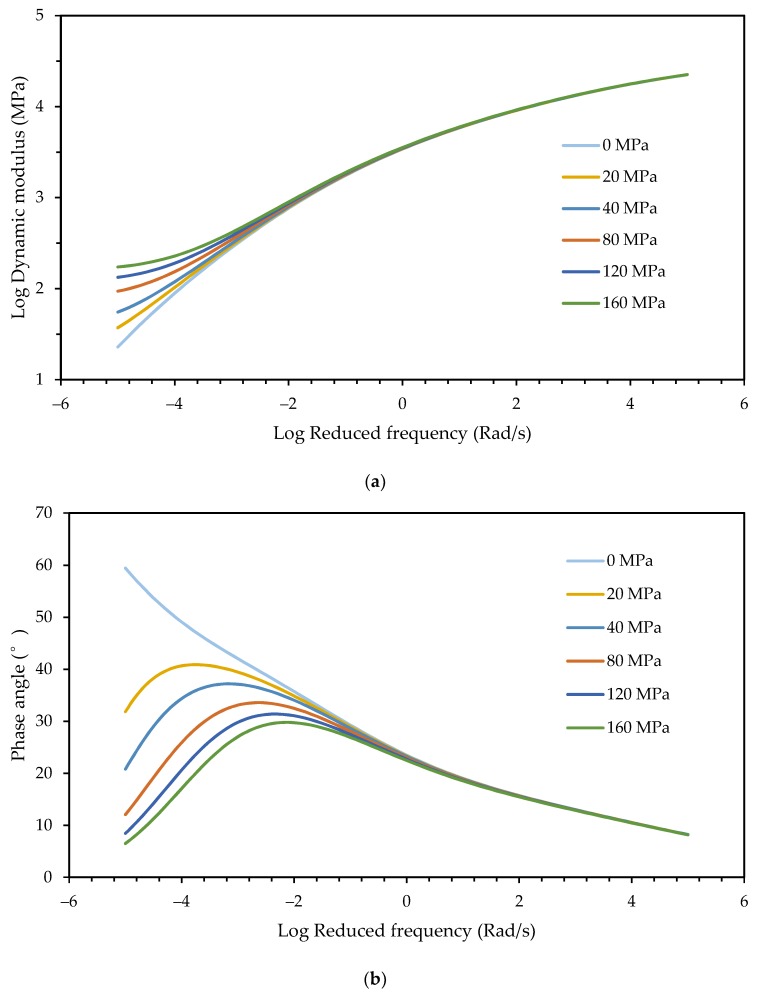
Effect of the equilibrium modulus: (**a**) Dynamic modulus; and (**b**) phase angle.

The effect of the equilibrium modulus on the master curves is shown in Figure 11.

It is seen that the equilibrium modulus had effects on both the dynamic modulus and phase angle at low frequencies. As the equilibrium modulus increased, the dynamic modulus increased and the phase angle decreased. The frequency at which the phase angle reached the peak also increased with the equilibrium modulus.

**Figure 12:**
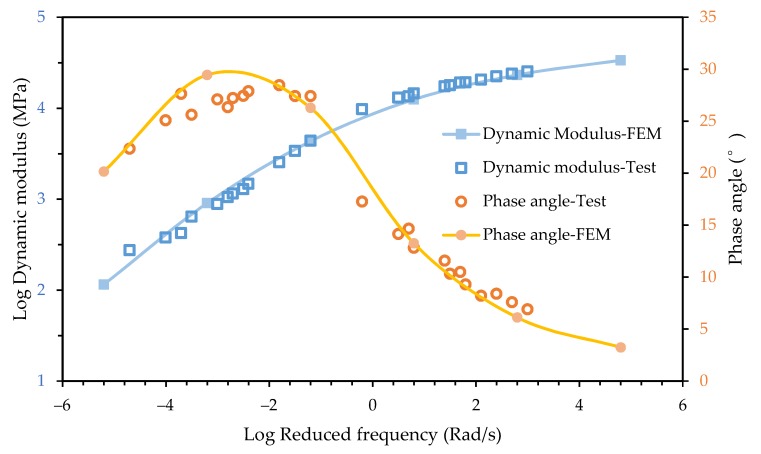
Improved prediction of the dynamic modulus and phase angle master curves.

In order to improve the accuracy of finite element simulation for the phase angle, the equilibrium modulus of the asphalt mixture was input into the finite element model as the equilibrium modulus of the asphalt mortar. Simulates with the same loading conditions as above were conducted and the results are shown in Figure 12.

It can be seen that the predicted phase angle was consistent with experimental data at all the frequency range. The increase of the dynamic modulus caused by the change of the equilibrium modulus at low frequency was not obvious. The simulated dynamic modulus was still in good agreement with the experimental data. It may be because that that the dynamic modulus of the mixture was mainly affected by the elastic modulus of aggregate at low frequencies. The result shows that the proposed method was effective to improve the prediction of the phase angle master curve. The finite element model could accurately predict the dynamic modulus and phase angle master curves of the asphalt mixture.

## 6. Conclusions

This study proposed a mesostructure-based finite element model of an asphalt mixture to predict both the dynamic modulus and phase angle master curves under a large frequency range. The difference between test data and simulation results of phase angle was observed. A new attempt was made to improve the accuracy of finite element simulation for the phase angle by modifying the equilibrium modulus of the asphalt mortar. Several conclusions can be drawn as follows:

(1) The 2S2P1D model originally proposed for the asphalt binder and asphalt mixture was used to characterize the viscoelastic mechanical response of the rubber modified asphalt mortar. The fitting results proved that the 2S2P1D model could accurately describe the dynamic modulus and phase angle master curves of rubber modified asphalt mortar. Furthermore, the continuous spectrum function of the 2S2P1D model was efficiently converted into a discrete spectrum for finite element implementation. The simulation results of the mesostructured-based finite element model showed that the discrete spectrum model could precisely predict the dynamic modulus of asphalt mixture under a large frequency range.
(2) The elastic modulus of aggregates demonstrated a stronger correlation with the phase angle of an asphalt mixture than the dynamic modulus. As the frequency increased, the effect on the dynamic modulus monotonically increased while the effect of the phase angle had fluctuated and reached a peak at 1 Rad/s.
(3) The test data of the phase angle indicated that the rubber modified asphalt mortar exhibited mechanical behavior of a viscoelastic liquid while the asphalt mixture was known as a viscoelastic solid. Then the mesostructure-based finite element simulation results proved that the prediction of the phase angle master curve strongly correlated with the mechanical behavior of the asphalt mortar. Therefore, a significant difference between test data and simulation results was observed at low frequency when directly using model parameters of the asphalt mortar. This difference was ignored by the existing studies in which simulations were conducted only under a small frequency range.
(4) The equilibrium modulus was found to have a great influence on the phase angle master curve through parameter sensitivity analysis. Further, simulation results proved that by replacing the equilibrium modulus of asphalt mortar with that of the asphalt mixture, the prediction of the phase angle could be significantly improved while the prediction of dynamic modulus was still acceptable.

In future studies, simulations for more types of asphalt mixtures with different asphalt binders and gradations need to be conducted to further verify the proposed method. The error fluctuations observed in Figure 10 need to be further analyzed. 

## Figures and Tables

**Table 1:** Specific surface area of aggregates.

Sieving Size (mm)	≥4.75	2.36	1.18	0.6	0.3	0.15	0.075
Specific surface area (m^2^/kg)	0.41	0.82	1.64	2.87	6.14	12.29	32.77

**Table 2:** Calculation of the asphalt content of the asphalt mortar.

Properties	Coarse Aggregates				Fine Aggregates					
Sieving size (mm)	16	13.2	9.5	4.75	2.36	1.18	0.6	0.3	0.15	0.075
Passing Ratio (%)	100.0	99.8	77.0	55.3	31.4	23.5	16.7	12.0	9.5	6.6
Specific surface area (m^2^/kg)	0.41	0.41	0.41	0.41	0.82	1.64	2.87	6.14	12.29	32.77
Surface area (m^2^)	0.00	9.34	8.88	9.81	6.49	11.10	13.43	15.47	35.40	217.59
Aggregate weight (kg)	68.36				31.41					
Surface area (m^2^)	28.03				299.49					
Proportion of surface area	0.09				0.91					
Coated asphalt (kg)	0.43				4.57					
Asphalt content (%)	12.71									

**Table 3:** Model parameters of the asphalt mixture and the asphalt mortar.

Materials	*E_e_* (MPa)	*E_g_* (MPa)	*δ*	*k*	*h*	*β*	*τ_ref_*	*C*_1_	*C*_2_	*T_ref_* (°C)	Relative Error
Asphalt mixture	127.33	53,993.61	2.62	0.08	0.45	57,370.2	0.07	82.04	804.57	0	4.96%
Asphalt mortar	0.00	40,166.80	5.45	0.20	0.52	1247.25	0.13	83.58	712.76	0	2.02%

## Appendix A

**Table A1:** The details of the Prony series.

$\mathrm{Log}\tau_{i}$ (s)	$E_{i}$ (MPa)	$\mathrm{Log}\tau_{i}$ (s)	$E_{i}$ (MPa)	$\mathrm{Log}\tau_{i}$ (s)	$E_{i}$ (MPa)	$\mathrm{Log}\tau_{i}$ (s)	$E_{i}$ (MPa)
−20.00	14.05	−13.05	354.98	−6.10	4570.31	0.85	1451.90
−19.57	17.86	−12.62	431.15	−5.67	4894.69	1.28	1115.67
−19.13	21.83	−12.18	522.85	−5.23	5139.13	1.71	816.32
−18.70	26.69	−11.75	632.90	−4.80	5284.67	2.15	568.91
−18.26	32.62	−11.31	764.38	−4.37	5320.12	2.58	379.64
−17.83	39.87	−10.88	920.67	−3.93	5244.20	3.02	244.40
−17.39	48.73	−10.45	1105.24	−3.50	5065.62	3.45	152.75
−16.96	59.53	−10.01	1321.51	−3.06	4801.28	3.89	92.83
−16.53	72.73	−9.58	1572.51	−2.63	4472.97	4.32	54.41
−16.09	88.82	−9.14	1860.37	−2.19	4103.59	4.75	29.95
−15.66	108.44	−8.71	2185.74	−1.76	3713.66	5.19	14.67
−15.22	132.34	−8.27	2546.96	−1.33	3318.95	5.62	5.97
−14.79	161.44	−7.84	2939.19	−0.89	2929.14	6.06	1.96
−14.35	196.82	−7.41	3353.53	−0.46	2547.93	6.49	0.54
−13.92	239.80	−6.97	3776.39	−0.02	2174.79	6.93	0.13
−13.49	291.91	−6.54	4189.42	0.41	1808.48	7.36	0.03
